# A Conditioned Response as a Measure of Impulsive-Compulsive Behaviours in Parkinson's Disease

**DOI:** 10.1371/journal.pone.0089319

**Published:** 2014-02-24

**Authors:** Andrew H. Evans, Jade Kettlewell, Sarah McGregor, Katya Kotschet, Robert I. Griffiths, Malcolm Horne

**Affiliations:** 1 Florey Neuroscience Institute, University of Melbourne, Parkville Victoria, Australia; 2 St Vincent's Hospital, Fitzroy, Victoria, Australia; 3 The Royal Melbourne Hospital, Parkville Victoria, Australia; 4 Department of Medicine, St Vincent's Hospital, Fitzroy, Victoria, Australia; Karolinska Institute, Sweden

## Abstract

**Objectives:**

Parkinson's Disease patients wore a device on the wrist that gave reminders to take levodopa and also measured bradykinesia and dyskinesia. Consumption of medications was acknowledged by placing the thumb on the device. Some patients performed this acknowledgement repeatedly and unconsciously. This study examines whether this behaviour reflected increased impulsivity.

**Methods and Results:**

Twenty five participants were selected because they had i) excess acknowledgements described above or ii) Impulsive-Compulsive Behaviours or iii) neither of these. A blinded assessor applied clinical scales to measure Impulsive-Compulsive Behaviours, cognition, depression, anxiety and apathy. A Response Ratio, representing the number of acknowledgements/number of doses (expressed as a percentage) was tightly correlated with ratings of Impulsive-Compulsive Behaviours (r^2^ = 0.79) in 19/25 subjects. Some of these patients had dyskinesia, which was higher with extraneous responses than with response indicating medication consumption. Six of the 25 subjects had high Impulsive-Compulsive Behaviour Scores, higher apathy scores, low levels of dyskinesia and normal Response Ratios. Patients without ICB (low RR) also had low dyskinesia levels regardless of the relevance of the response.

**Conclusion:**

An elevated Response Ratio is a specific measure of a type of ICB where increased incentive salience is attributed to cues by the presence of high striatal dopamine levels, manifested by high levels of dyskinesia. This study also points to a second form of ICBs which occur in the absence of dyskinesia, has normal Response Ratios and higher apathy scores, and may represent prefrontal pathology.

## Introduction

There is increasing recognition of Impulsive-Compulsive Behaviours (ICBs) in people with Parkinson's Disease (PD) [1]–[6] that include pathological gambling, compulsive shopping, hypersexuality, binge eating, punding, hobbyism, hoarding[7], kleptomania[8], impulsive smoking[9] and compulsive medication use. In clinical practice, ICBs are recognised by astute clinical enquiry and self-reporting, but many patients fail to report ICBs because of embarrassment and lack of awareness of the relationship between PD and ICBs[10]. The neurobiological mechanisms of ICBs in PD are under active investigation. One model posits that ICBs reflect an aberrant form of learning. During Pavlovian learning, previously neutral stimuli that predict rewards can acquire motivational properties, becoming attractive and desirable incentive stimuli. However, individuals may vary in the extent to which a cue acts solely as a predictor of reward, or also serves as an incentive stimulus. Thus, individuals vary in the degree to which cues bias choice and potentially promote maladaptive behaviour. One proposal is that dopamine acts selectively in a form of stimulus-reward learning in which incentive salience is assigned to reward cues [11]. On the other hand, other authors suggest that in PD, dopamine has a more global role in sensitization to a range of appetitive behaviours in vulnerable individuals[12], which implies that PD patients with ICBs might be more sensitive to forms of stimulus-reward learning in which incentive salience is assigned to “reward” cues.

Recently we identified a group of PD patients who appeared to overuse a trained cue to acknowledge a medication intake. These patients were using a system for long term (10 day) measurement of bradykinesia and dyskinesia in people with PD [13]. The system includes a facility for programming the timing of levodopa into a wrist worn device, which then delivers a vibration to the wrist to remind the patient to take medications. Patients were trained to acknowledge a medication intake by placing their thumb on the device and, after a short time delay, removing the thumb during a brief acknowledgement signal. Most patients were highly compliant but a proportion activated the acknowledgement signal repeatedly, especially in the afternoons. This was not in recognition of intake of extra medication, but was an unconscious and spontaneous process. We therefore speculated that these patients who appear to overuse a trained cue to acknowledge a medication intake might be an example of increased sensitivity to stimulus-reward learning in which incentive salience is assigned to “reward” cues.

The study described here was to establish whether overuse of this acknowledgement system was a marker of ICB presence. We found that people with PD who had ICBs, used the acknowledgment excessively, especially when dyskinetic. We discuss the possibility that this arises because high striatal dopamine associated with dyskinesia increases the propensity to abnormally assign “incentive salience” to reward cues[11], which in this instance are the response to medications.

We aimed to establish whether overuse of this acknowledgement system was a marker of ICB presence and whether overuse of this acknowledgement was predicted by variables previously reported to predict ICBs such as dyskinesia[4], [14].

## Materials and Methods

This study, including the consent form was approved and supervised by the St Vincent's Hospital Human Research & Ethics Committee. All subjects provided written consent. Subjects with idiopathic levodopa responsive PD were recruited from the Movement Disorder Clinic at St Vincent's Hospital.

### Recording Protocol

The Parkinson's Kinetigraph data logger[13] (PKG, Global Kinetics Corp. Aus) was used for recording bradykinesia and dyskinesia[13]. This logger is worn on the wrist of the side of the body most severely affected by Parkinsonism and contains a rechargeable battery, a triaxial accelerometer, memory, a mechanism for programming medication reminders and recording the timing of medications. The logger can detect whether it is being worn and although patients were asked to wear it continuously for 10 days, only those periods when it was worn were analysed in this study.

At the end of the recording period, data was downloaded and analysed by a proprietary algorithm to calculate a bradykinesia score (BKS) and a dyskinesia score (DKS)[13]. This algorithm and the PKG system has been described in a previous publication[13]. The time of day when levodopa medications were prescribed can be programmed into the data logger and used to generate a reminder, consisting of an 11 s vibration (duty cycle: 400 ms on/800 ms off) applied to the wrist at the prescribed time. The subject marks the consumption of medication by placing their thumb on the logger for 3 seconds, which is “acknowledged” by the logger with a brief vibration (1 s). These are described as “expected” responses to distinguish them from “unexpected” acknowledgements occurring without a reminder. An unexpected acknowledgement is also produced by placing the thumb on the logger for 3 seconds, when a light appears on the logger. The patient then must remove their thumb within 1.75 s of the light appearing. If this process is successfully completed then a brief acknowledging vibration occurs. This is referred to as an “expected response”. If the same process of holding the thumb in place for 3 seconds, followed by the acknowledgement light was repeated, but without a preceding reminder then only a brief (1 s) vibration was triggered. This is referred to as an “unexpected response”. Briefer or longer “touches” to the logger's detection area were neither acknowledged or recorded. Thus, inadvertent activation by excessive movement (as in dyskinesia) or by posture was unlikely.

### Calculation of the Response Ratio to reminders to take medications

**Figure 1:**
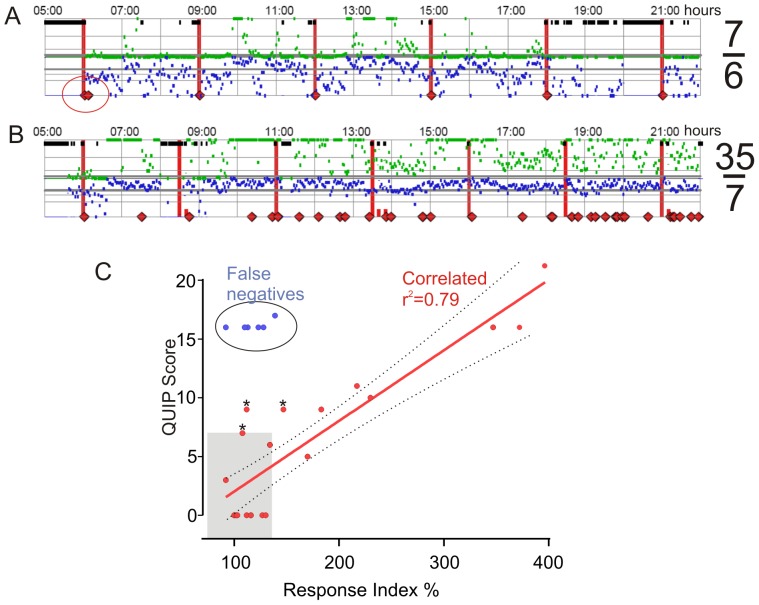
The Response Ratio. Fig. 1A. Example of the output from one day of PKG recording from a patient who was prescribed 6 doses of levodopa/day. The green and blue dots represent the dyskinesia and bradykinesia (respectively) score, which was calculated every 2 minutes, with greater severity represented by increasing distance from the middle of the graph. The horizontal lines are the medians, 75^th^ percentile and 90^th^ percentiles of controls. The red vertical lines are when medications were prescribed and the diamonds are when the taking of medication was acknowledged. This subject provided a second acknowledgment to the 6:00 am dose shortly after the first acknowledgement (circle), thus providing 7 acknowledgments for 6 doses. The response ratio (RR) for this patient was 116% (7/6). Fig. 1B. This subject was prescribed 7 doses/day but provided 35 acknowledgements. Note that this subject had many more dyskinesia scores at higher levels with many at the upper levels of the graph (e.g. 10:00 am to 11:00 am). The response ratio (RR) for this patient was 500% (35/7). Fig. 1C: A plot of the QUIP score (y axis) plotted against the RR (X axis). Two groups are apparent: False Negatives (blue dots: n = 6), and a Correlated Group (Red dots: n = 19). The grey shaded area represents people whose RR was less than 137% and whose ICB score was 7.

Subjects were instructed to take medications upon receiving the reminder from the PKG logger and to then immediately acknowledge their consumption as described above. The subject in Fig. 1A is typical, in that the reminder (red vertical bar) is closely associated with the red diamond (acknowledgment). This subject received 6 reminders and gave 7 acknowledgements and this was expressed as a Response Ratio (RR) where the total number of responses (expected and unexpected) is expressed as a percentage of the number required (expected). Thus the RR for the subject in Fig. 1A was 116. The patient in Fig. 1B, however, provided 35 acknowledgments when only 7 were expected, expressed as an RR of 500. The RR was calculated for the whole 10 days that the logger was worn.

### Patient Selection

The median RR in 108 subjects (mean age 64 years, disease duration 8 years) who had worn the PKG in the past 6 months as part of routine clinical care was 116 and the 75^th^ percentile was 136, which was taken as the upper level of a normal RR for subsequent analyses in this study. A subset of 30 of these 108 subjects were further selected because their median DKS was greater than the median of controls. Subjects were chosen by an unbiased, first-come, first-taken search.

The main study was undertaken on another 25 subjects. Six of these subjects were identified from a review of the clinical records as subjects who had not worn the PKG, but were known to have high QUIP scores (ie at least one from Category A, B or C in the present and at least one from the past). As well 19 of the 108 subjects, whose ICB status was unknown, were selected because they either had a high RR (>137: n = 7) or because their RR was less than 137 (n = 12). These cases were not known to the selector and were chosen by going backwards in time through the records until a study cohort of 25 was obtained.

### Blinded Questionnaires

A blinded examiner administered the Starkstein Apathy scale (AS) [15] and the Questionnaire for Impulsive-Compulsive Disorders in Parkinson's disease (QUIP)[16]. QUIP scoring was modified into a numerical scale by scoring Category A as six, Category B as five and Category C as four if they were current and as three, two and one respectively if the were some time in the past. The United Parkinson's Disease Rating Scale (parts 1–4)[17], the Scales for Outcomes in Parkinson's disease-Cognition (SCOPA-COG)[18], the Addenbrooke's Cognitive Assessment- Revised (Australian Version)[19] were all performed in the “on” state. Anti-Parkinson's Medications including the total levodopa equivalent dose (LED) and dopamine receptor 2 agonist use, age and age of onset of disease were all recorded.

### Self-reporting Questionnaires

Patients were instructed to complete the Beck Depression Inventory (BDI)[20], BIS/BAS (Behavioural Inhibition Scale/Behavioural Activation Scale)[20] and State Trait Anxiety Inventory (STAI)[18] in isolation. The BDI was used to assess if the subject suffers from depression[20] and the STAI used to indicate the presence of state and/or trait anxiety[21]. The BIS/BAS questionnaire was scored using the BIS/BAS scale (reverse scoring apart from questions 2 and 22) and the total score was split into 4 parts; BIS, BAS drive, BAS fun seeking and BAS reward responsiveness.

### Statistics

Most data was not normally distributed and consequently, data is presented as median and interquartile range and non parametric tests (e.g. Mann-Whitney) were used with p<0.05 used as a measure of significance. When ANOVA was required a Kruskal Wallis test was used and the Mann-Whitney was used to the specific sample pairs for significant differences.

## Results

### Response to reminders to take medications

The RR in the 108 subjects who had worn the PKG as part of routine clinical care was examined. Twenty three percent of patients who took 4 or less dose of levodopa/day had RR scores over the 75^th^ percentile (136) whereas 63% taking 5 or more doses/day (p<0.05 χ^2^ test). This indicates that high RRs are more common in patients with more frequent levodopa dosing. On the other hand the 30/108 subjects with a high median DKS (dyskinesia score) did not have a greater tendency to have a high RR (Figure S1) arguing that a high RR was not due to dyskinesia *per se* or brought about by inadvertent mechanical activation of the acknowledgement sensor.

### RR and QUIP scores

The possibility that the increased RR may represent a form of ICB was investigated by examining the relationship between RR and modified QUIP, BIS/BAS, STAI and Starkstein AS scores in 25 subjects. A second assessor, who was blinded to the selection process, administered the scales and questionnaires and arranged for all subjects to wear a PKG. The modified QUIP score was plotted against the RR score obtained from the recent PGK report (Fig. 1C). In 17 subjects there was a strong correlation (r^2^ = 0.79) with modified QUIP scores but 6 were clear outliers and fell into False Negative group (Fig. 1C). Thus, the RR was a good predictor of the severity of ICB, as measured by modified QUIP in 75% of subjects (correlated group) but 25% were false negatives: they had high modified QUIP scores but a RR within the normal range.

### Difference between Correlated Group and False Negative Group

**Figure 2:**
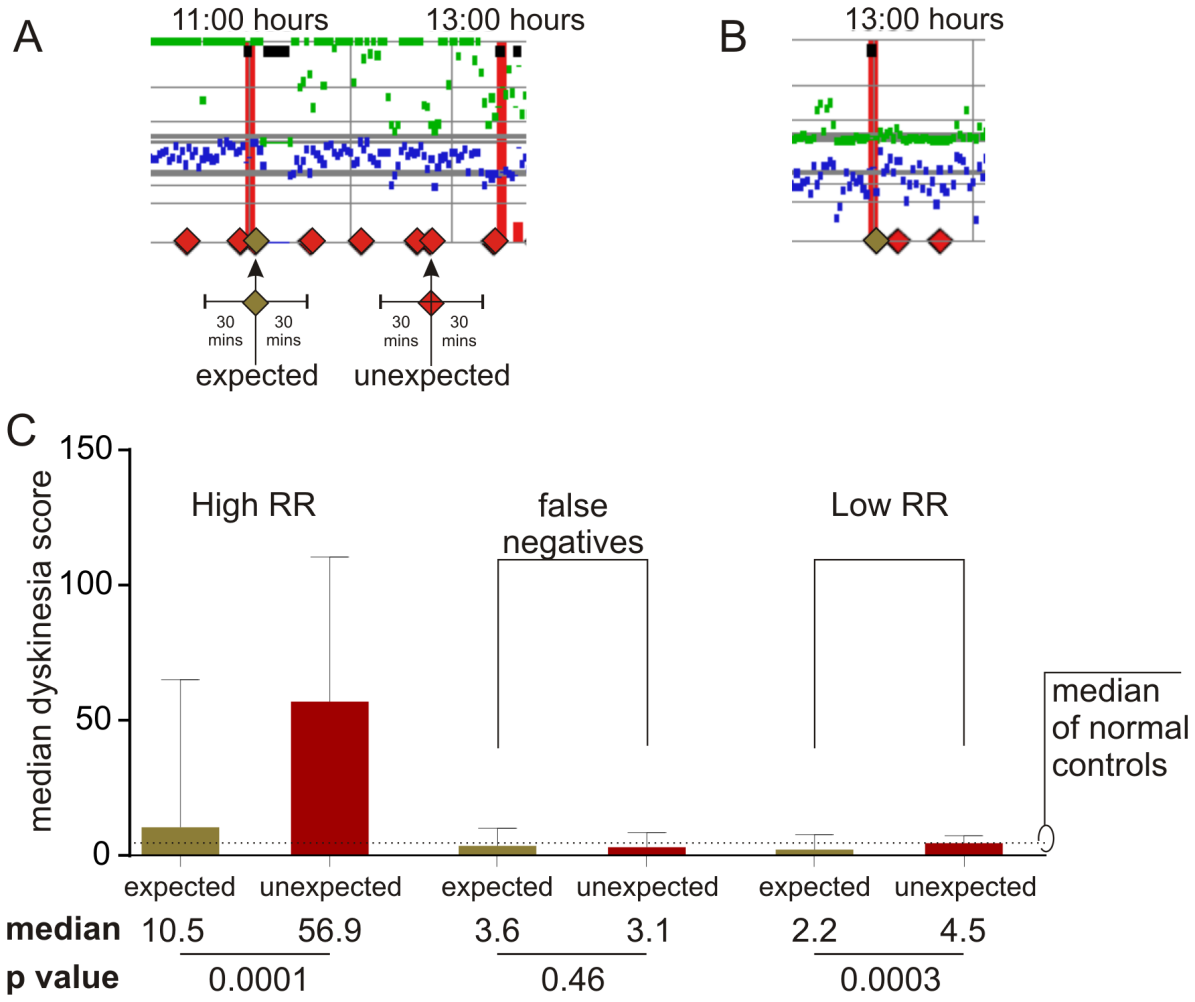
Dyskinesia in the 30 minutes around each response. Fig. 2A. A segment of the daily PKG record from a person with ICB, showing expected responses (tan diamonds) and unexpected responses (red diamonds). Note that in this example, the 2 minute dyskinesia scores are mostly high. Fig. 2B. A segment of the daily PKG record from a person without ICB, showing expected responses (tan diamonds) and unexpected responses (red diamonds). Note that in this example, the 2 minute dyskinesia scores are below the median for controls. Fig. 2C. Histograms of the median dyskinesia score in the 30 minutes either side of expected (tan bars) and unexpected (red bars) response in the High RR group, the False Negative Group and in low RR Group. The histogram bars represent the median and 75^th^ percentile values for the subjects in each group. The median dyskinesia score in control (i.e. non PD) subjects is shown as a dotted line. Note that median dyskinesia scores of both expected and unexpected responses in the False Negative Group and the low RR subjects were below the median of controls. On the other hand, while the dyskinesia scores associated with expected responses in the correlated group were high, they were significantly higher still in the period surrounding unexpected responses. The P values associated with expected and unexpected response is shown.

Because ICBs have been associated with development of dyskinesia, we examined dyskinesia scores generated by the PKG in the False Negative Group, a high RR Group (RR>136) and a low RR group (RR<137) subjects. First, the median of the 2 minute dyskinesia scores from the PKG in the 30 minutes either side of each response (acknowledgement) was calculated (Fig. 2 A and 2B). Then the median dyskinesia score of the expected acknowledgments after a reminder was compared with median dyskinesia score at the time of the unexpected responses (that led to increased RRs, Fig. 2C). The median dyskinesia scores of both expected and unexpected responses in the False Negative Group and the low RR subjects were below the median of controls (Fig. 2C). In comparison, the dyskinesia scores in the 30 minutes either side of expected responses in the correlated group were high, but were significantly higher still in the period surrounding unexpected responses (Fig. 2C).

The False Negative Group tended to have higher Starkstein Apathy scores than the Correlated Group (p = 0.06 Mann Whitney) and a less significant trend toward higher STAI (p = 0.15 Mann Whitney) and lower BIS/BAS scores (p = 0.16 Mann Whitney). Taken together this suggests that the False Negative Group are a separate entity who have ICBs without dyskinesia and have higher apathy but lower impulsivity than might otherwise be expected of subjects with ICBs.

**Figure 3:**
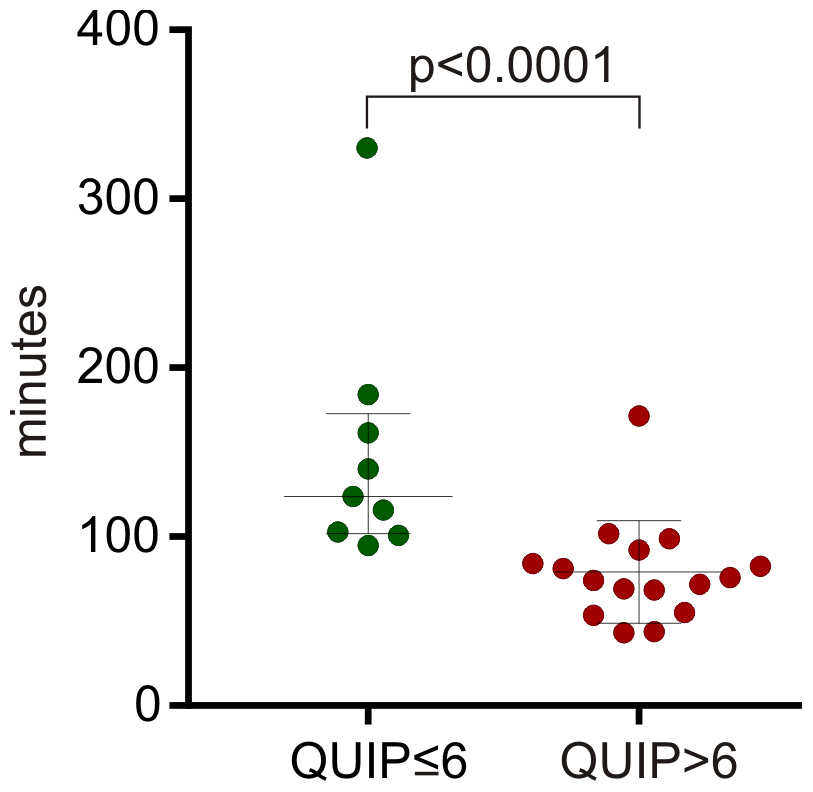
Latency of unexpected responses from the most recent levodopa dose. The median latency (in minutes, Y axis) of unexpected response from the most recent dose of levodopa in patients whose QUIP scores were >6 or ≤6. The median latency of unexpected responses was 75 minutes (interquartile range 59–90 minutes) in those with higher QUIP scores compared to 123 minutes (interquartile range 101–173 minutes) in those with low QUIP scores (p<0.0001: Mann-Whitney).

If the unexpected responses were related to high levels of striatal dopamine, then their timing with respect to the most recent consumption of levodopa would be of interest. For each patient, the time (latency) of each unexpected response from the most recent scheduled levodopa dose was calculated. Then for each patient the median latency of all unexpected response was calculated and plotted against QUIP score, which was well correlated (r^2^ = 0.68, data not shown). Patients were then sorted according to whether the QUIP score was >6 or ≤6 (Figure 3). The median latency of unexpected responses was 75 minutes (interquartile range 59–90 minutes) in those with higher QUIP scores compared to 123 minutes (Interquartile range 101–173 mins) in those with low QUIP scores (p<0.0001: Mann-Whitney).

### RR and Motor Scores

The duration of PD in the High RR group was longer (medians 16 v 10 years: p<0.05 Mann Whitney) and the BDI scores (medians 15 v 7 years: p<0.05 Mann Whitney) higher than the low RR group (Table 1). The values for the False Negative subjects fell between the other two groups and did not reach significance (Table 1). There was a trend for the high RR group to have worse UPDRS, ACE-R, SCOPA COG, STAI and AS scores and to take more LED, but only the LED (P<0.05 Mann Whitney) reached statistical significance (Table 1). The values in the False Negative group were in general intermediate between the other two groups. One third of the Low RR group (4/11) were on dopamine receptor 2 agonists whereas 2/8 of the High RR subjects and 5/6 of the False Negatives were on dopamine receptor 2 agonists.

**Table 1:** Clinical Demographics and performance on Clinical Rating Scales.

Percentile	Age	Durat'n	LED	UPDRS					ACE	SCOPA	BDI	BIS/	STAI	AS
	Yrs	Yrs		1	2	3	4	Total	-R	-COG		BAS		
**Low RR**														
25^th^	51	6	625	0	6	7	2	17	91	26	6	66	53	5
50^th^	73	10	874	2	8	14	4	33	96	28	7	70	63	7
75^th^	76	14	1225	3	13	23	7	40	97	32	10	77	86	11
**High RR**														
25^th^	64	14	1106	1	11	9	6	33	86	23	8	73	60	3
50^th^	69	16	1813	3	12	19	8	44	91	30	15	77	77	7
75^th^	75	22	2529	5	17	28	10	53	95	32	21	79	105	17
**False Neg**														
25^th^	61	5	982	1	13	14	3	32	84	22	8	63	78	8
50^th^	64	9	1300	2	14	18	6	39	94	29	10	72	82	11
75^th^	75	13	1975	4	20	40	8	73	94	35	13	79	98	14

See text for abbreviations.

The median dyskinesia score from the PKG obtained from 10 days of recording in the time between 9:00 and 18:00 was significantly higher in the high RR group than in the low RR and False Negative groups (P<0.05: Mann Whitney) as were the UPDRS IV scores (P<0.05: Mann Whitney). The median dyskinesia scores in the False Negative Group were intermediate between the Correlated and non ICB groups.

### The discriminatory value of the RR as a screen for ICBs

Fig. 1C indicates that the False Negatives are outliers, lying well outside the confidence limits of the other subjects and the data, presented above, points to grounds for regarding them as a separate entity. In this context it is interesting to speculate whether the three red dots marked by asterix, which fall outside the confidence limits, are indeed incipient members of the False Negative Group. Most of the subjects in the study were questioned about the reasons that they used the reminders excessively. Many were unaware that they had responded excessively and those who were aware could not provide an explanation. All were adamant that they did not use it to indicate increased consumption of medications. When the False Negative group are excluded, there was high level of agreement between the modified QUIP score and RR Index in detecting ICBs (p = 0.0012: Fishers exact and free-marginal *kappa* value[22] of 0.79).

## Discussion

This study shows that a high RR index is a sensitive test for the presence of ICBs. In other words, subjects with a High RR will almost always have ICBs. The question of whether the failure to detect the False Negative Group indicates reduced selectivity or instead, a separate form of ICBs will be discussed below. The findings that subjects with high RR/modified QUIP scores in the correlated group had higher median dyskinesia scores over the ten days while wearing the PKG is in keeping with previous reports that ICBs are commonly found in subjects with dyskinesia[23]. Indeed, risk factors for dyskinesia and fluctuations are the same for developing ICBs[23]–[27] and include disease severity, disease duration, daily dose of levodopa, and age at onset[28]–[31]. The findings that the UPDRS IV was also higher in this group supports the PKG data, but also reflects the greater sensitivity of continuous objective measurement of dyskinesia with the PKG in evaluating dyskinesia. However further studies [13] supporting the relationship between the PKG's measurement of dyskinesia and conventional scales would strengthen this argument.

ICBs are also more likely to occur in the ”on” state in those at risk of ICBs[32]. Thus the finding that dyskinesia scores are high in the 30 minutes either side of an expected response is not surprising: but it is intriguing that the dyskinesia scores at the time of unexpected responses are higher still and raises the possibility that the unexpected responses reflect a disturbance in the reward related processes. Dopamine in the striatum is central to this process[33], [34] and dopamine levels are excessively high in the putamen during dyskinesia[35]. Medication induced neuroplastic changes leading to elevated ventral striatal dopamine has also been invoked to explain the development of ICBs of PD and may be linked to compulsive drug wanting in patients with the dopamine dysregulation syndrome[12], [36]. ‘Learning’ models of addiction suggest that drugs promote the learning of strong ‘automatized’ stimulus–response (S–R) habits and rituals involved in consuming drugs[37]. Moreover, in animals, sensitization of dopaminergic systems promotes S-R processes and subsequently increases the control of behaviour by reward- related cues[38], perhaps via recruitment of the dorsal striatum[39]. However, the behaviours examined here (which consist of a tactile stimulus, followed by ingestion of medications that cause excessively elevated levels of dopamine in the striatum), may represent more than an enhanced ‘learning’ of an acknowledgement response. Rather than reflect more than dominance of S-R habits resulting from a sensitized dopaminergic systems, we propose that the Response has taken on a salience similar to that of a cue in a conditioned stimulus in associative learning. Recently it was demonstrated that in some subjects, striatal dopamine may contribute to the attribution of Pavlovian incentive values to cues that signal reward, thus making them valuable in their own right[11], [40]. Individuals with a propensity to this form of reward learning, where “incentive salience” is assigned to reward cues, are at risk of the cues driving the behaviour[[11]. However, where the cue does not carry this incentive salience, striatal dopamine does not play the same central role in the associative learning. We propose that Correlated Group represent a group, with abnormally high levels of striatal dopamine, where the Response has gained salience in its own right. It is a marker for ICBs because it is a marker of the risk of propensity to abnormally assign “incentive salience” to reward cues. Conversely, the False Negative group may represent a different form of ICB. Recent data emerging from the addiction literature emphasizes the role of changes in the function of frontal circuitry associated with the overvaluing of drug reinforcers, the undervaluing of alternative reinforcers, and deficits in inhibitory control for drug responses[41] where dopamine may play less of a role. Similar frontal deficits have been reported in PD patients with apathy[42]] and suggests that further examination of this group is required.

If an increased RR reflects high levels of dopamine in the striatum causing both dyskinesia and impulsivity, then it might not be surprising that dyskinesia scores were high. In these patients, dyskinesia scores were high even in the 30 minutes surrounding an expected response, albeit lower than when other responses occurred. ICBs are more likely to occur at the time of dyskinesia[23], whereas anxiety and dysphoria anticipates wearing off [43]. Furthermore, in Dopamine Dysregulation Syndrome mood and anxiety also fluctuates with motor state, especially as DA levels fall[44]. In particular, the end of a levodopa dose is associated with rebound worsening in aspects of affect in dyskinetic subjects[43]. The Dopamine Dysregulation Syndrome [44], [45] and probably the Dopamine Agonist Withdrawal [46] are driven more by a dysphoric state drug “wanting” and reward responsivity associated with falling DA levels[44]. These patients take more dopamine replacement therapy, had worse sleep and were more anxious and depressed[27]. This may be analogous to the situation in which compulsive drug wanting and change affect is linked to ventral striatal dopamine function[36] whereas change in UPDRS III has been linked to dorsal striatal dopamine systems[47]. The data in Figure 3 suggest that the unexpected responses occur between 60 and 90 minutes after a levodopa dose in patients with high QUIP scores, at a time when plasma levels should be peaking. When “wearing off” and dyskinesia are well established, the duration of efficacy of a single dose of levodopa mirrors the plasma levels of levodopa[28], [48]–[50]. Thus the data in Figure 3 indirectly implies that unexpected responses occur at the time when DA levels peak in the striatum.

While patients denied excess consumtion, we cannot exclude the possibility that some patients took extra doses and the high dyskinesia levels simply reflect this increased consumption which was acknowledged by the extra responses. However most patients seemed genuinely unaware of making excess acknowledgements and it seems unlikely that they would deny excess doses while at the same time acknowledging their consumption on the logger. Furthermore, the rate of responses of many patients would reflect very high doses: the patient in Fig. 1B would have taken 7000 mg (35×200 mg) of levodopa/day. Furthermore it seems implausible that patients would both deny increase consumption yet at the same time diligently acknowledge each extra dose through the PKG mechanism. Thus while increased consumption can be categorically excluded, it seem unlikely to be the main explanation for the association between high RR and dyskinesia.

In summary an elevated Response Ratio is a sensitive measure of ICBs that are linked to increased dyskinesia. We believe that this type of ICB represents the attribution of increased incentive salience attributed to cues by the presence of high striatal dopamine levels. This study also points to a second form of ICBs which occur in the absence of dyskinesia, have normal RR and who have trend to higher apathy scores. This second form may represent prefrontal pathology.

## Supporting Information

Figure S1 **Thirty subjects whose median DKS (dyskinesia score) was greater than the median of controls was selected from the 108 subjects.** Subjects were taken in order from the list without a bias. Their RR (y axis) was plotted against the median DKS (x axis). The Black square on the Y axis and the associated dotted line represent the median of controls. A high RR was not associated with a high DKS, arguing that a high RR was not due to dyskinesia *per se* or brought about by inadvertent mechanical activation of the acknowledgement sensor.
(TIF)
