# Supplementary figures and images for: A Conditioned Response as a Measure of Impulsive-Compulsive Behaviours in Parkinson's Disease
Source: PLoS One. 2014 Feb 24;9(2):e89319. doi: 10.1371/journal.pone.0089319 (PMC3933354; doi:10.1371/journal.pone.0089319)

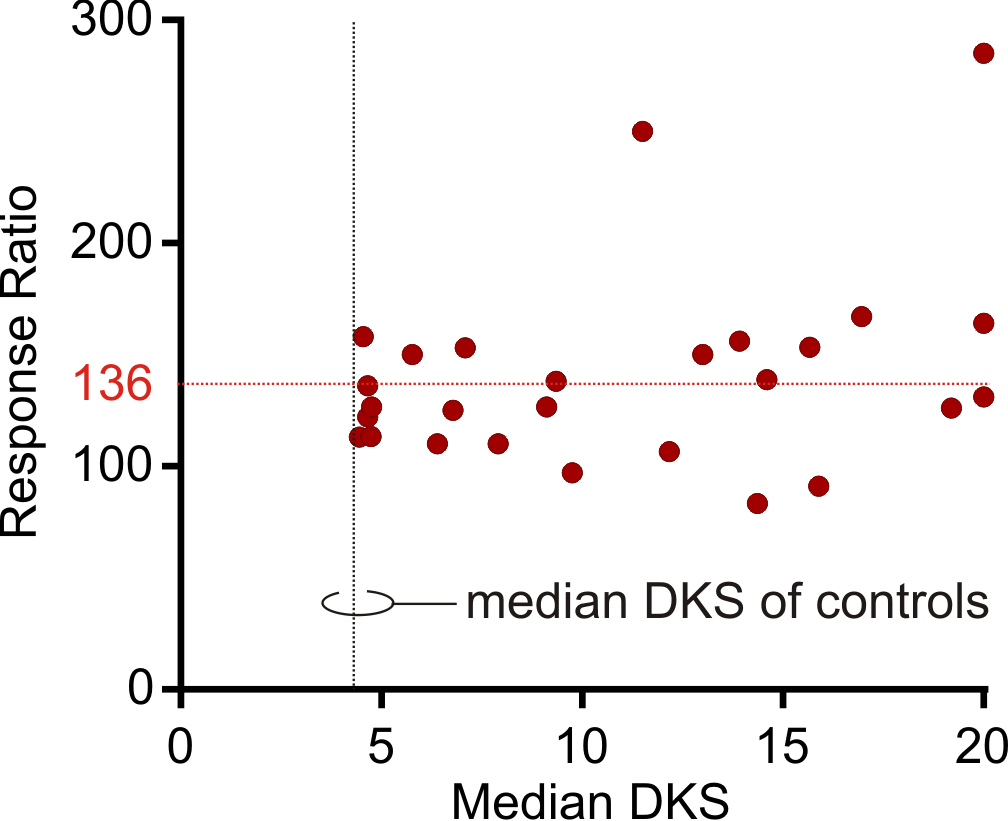

Supplement: Figure S1 — Thirty subjects whose median DKS (dyskinesia score) was greater than the median of controls was selected from the 108 subjects. Subjects were taken in order from the list without a bias. Their RR (y axis) was plotted against the median DKS (x axis). The Black square on the Y axis and the associated dotted line represent the median of controls. A high RR was not associated with a high DKS, arguing that a high RR was not due to dyskinesia per se or brought about by inadvertent mechanical activation of the acknowledgement sensor. (TIF) [file pone.0089319.s001.tif]
